# The Role of Class IA Phosphatidylinositol-4,5-Bisphosphate 3-Kinase Catalytic Subunits in Glioblastoma

**DOI:** 10.3389/fonc.2017.00312

**Published:** 2017-12-15

**Authors:** Kevin J. Pridham, Robin T. Varghese, Zhi Sheng

**Affiliations:** ^1^Virginia Tech Carilion Research Institute, Virginia Tech, Roanoke, VA, United States; ^2^Graduate Program in Translational Biology, Medicine, and Health, Virginia Tech, Blacksburg, VA, United States; ^3^Edward Via College of Osteopathic Medicine, Blacksburg, VA, United States; ^4^Virginia Tech Carilion School of Medicine, Virginia Tech, Roanoke, VA, United States; ^5^Department of Biomedical Sciences and Pathobiology, Virginia-Maryland College of Veterinary Medicine, Virginia Tech, Blacksburg, VA, United States; ^6^Faculty of Health Science, Virginia Tech, Blacksburg, VA, United States

**Keywords:** phosphatidylinositol-4,5-bisphosphate 3-kinase, glioblastoma, class IA phosphatidylinositol-4,5-bisphosphate 3-kinase catalytic subunits, PIK3CA, PIK3CB, PIK3CD

## Abstract

Phosphatidylinositol-4,5-bisphosphate 3-kinase (PI3K) plays a critical role in the pathogenesis of cancer including glioblastoma, the most common and aggressive form of brain cancer. Targeting the PI3K pathway to treat glioblastoma has been tested in the clinic with modest effect. In light of the recent finding that PI3K catalytic subunits (PIK3CA/p110α, PIK3CB/p110β, PIK3CD/p110δ, and PIK3CG/p110γ) are not functionally redundant, it is imperative to determine whether these subunits play divergent roles in glioblastoma and whether selectively targeting PI3K catalytic subunits represents a novel and effective strategy to tackle PI3K signaling. This article summarizes recent advances in understanding the role of PI3K catalytic subunits in glioblastoma and discusses the possibility of selective blockade of one PI3K catalytic subunit as a treatment option for glioblastoma.

## Introduction

### Glioblastoma

Glioblastoma is a grade IV glioma accounting for approximately 55% of all cases of malignant brain tumors. The 5-year overall survival for glioblastoma is only about 5% even after aggressive treatments including maximal surgical removal of the tumor, ionizing radiation, and chemotherapy (1–6). This poor prognosis is, at least in part, owing to the high incidence of tumor recurrence; nearly 100% of glioblastoma patients will succumb to tumor recurrence if they live more than 2 years. The average survival for patients with recurrent glioblastoma is about 7.5 months (7), because these patients often cannot undergo another major resection, and the tumors are resistant to radiation and chemotherapies (7–10). With no standard of care treatment for recurrent glioblastoma, new strategies for therapeutic intervention are urgently needed. Therapies targeting essential survival pathways in glioblastoma [e.g., inhibitors of receptor tyrosine kinases (RTKs) or signaling molecules] have achieved modest, yet encouraging, therapeutic benefits in recurrent glioblastoma (11–22). The difficulty of designing targeted therapies in the clinic is often attributed to the lack of biomarkers for patient selection, the high toxicity of these drugs, the difficulties in delivering drugs into the brain, and the unclear mechanisms of therapeutic targets in glioblastoma.

### Phosphatidylinositol-4,5-Bisphosphate 3-Kinase (PI3K) Signaling

The PI3K pathway plays an essential role in signal transduction and modulates various biological processes including cell proliferation, survival, motility, death, and metabolism (23, 24). Aberrations in these processes are pivotal in the pathogenesis of cancer, including glioblastoma (25, 26). Upon activation of RTKs or G protein-coupled receptors (GPCRs), PI3K phosphorylates phosphatidylinositol-4,5-bisphosphate (PIP2 or PtdIns4,5P_2_) on the plasma membrane, yielding phosphatidylinositol-3,4,5-triphosphate (PIP3 or PtdIns3,4,5P3), which subsequently activates V-Akt murine thymoma viral oncogene homolog (AKT) to inhibit cell death and thereby sustain cell survival. The PI3K pathway is negatively regulated by the tumor suppressor phosphatase and tensin homolog (PTEN), which dephosphorylates PIP3 [reviewed in Ref. (27, 28)]. Based upon genome sequencing data from The Cancer Genome Atlas (TCGA), more than 88% of glioblastoma tumors harbor mutations in the signaling pathways driven by RTKs, oncogenic RAS gene family, and/or PI3K (29). PTEN is altered in approximately 40% of glioblastoma patients and is considered as a biomarker of glioblastoma prognosis (30, 31). Frequent mutations in PTEN and certain PI3K genes have been reported in primary and recurrent glioblastomas (29, 32–40). The oncogenic role of these genes has been validated in genetically engineered mouse models of glioblastoma [summarized in Ref. (41–43)]. A recent study analyzing the spatiotemporal genomic architecture of glioblastoma found that mutations of a PI3K gene were commonly clonal, early events of tumorigenesis, and affected the tumor response to therapies (44).

Because of the critical role of hyper-activation of PI3K in cancer and therapeutic resistance, considerable efforts have been directed to develop chemical inhibitors targeting the PI3K/AKT signaling pathway. To date, there are more than 50 chemical compounds specifically blocking the activity of PI3K/AKT and showed promising effects on tumor inhibition in preclinical studies, but only some drugs have successfully entered clinical trials for glioblastoma treatment (Table 1). PI3K or PI3K/mechanistic target of rapamycin (mTOR) dual inhibitors (e.g., XL765, wortmannin, PI-103, PX-866, and LY294002) alone or in combination with radiotherapy or chemotherapy substantially inhibited glioblastoma cell growth in preclinical studies (45–49). A pan PI3K inhibitor PX-866 showed modest effect on the prognosis of 33 recurrent glioblastoma patients in a phase II clinical trial (12). While this is encouraging, the high toxicity and severe side effects associated with non-selective PI3K inhibitors has limited their clinical applications (50–53). An in-depth understanding of PI3K signaling will help improve the therapeutic efficacy of PI3K-based glioblastoma therapies.

**Table 1:** Phosphatidylinositol-4,5-bisphosphate 3-kinase (PI3K) inhibitors in clinical trials for glioblastoma in the United States.

PI3K inhibitors	Targets	Trial phases	NCT number
XL765	PI3K/mechanistic target of rapamycin (mTOR)	I	NCT01240460
XL147	Pan-PI3K	I	NCT01240460
XL765	PI3K/mTOR	I (with temozolomide)	NCT00704080
BEZ235	PI3K/mTOR	I/II	NCT02430363
GDC-0491	Pan-PI3K	I/II	NCT02430363
BKM120	Pan-PI3K	II	NCT01339052
BKM120	Pan-PI3K	I/II (with INC280)	NCT01870726
BKM120	Pan-PI3K	I/II (with Bevacizumab)	NCT01349660
BKM120	Pan-PI3K	I (with LDE225)	NCT01576666

*Data were retrieved from http://clinicaltrials.gov. Temozolomide is a DNA alkylating agent used for treating glioblastoma. INC280 is an inhibitor of MET proto-oncogene. Bevacizumab targets vascular endothelial growth factor. LDE225 is an inhibitor of Hedgehog signaling*.

## The Divergent Roles of Class IA PI3K Catalytic Subunits in Glioblastoma

### Classification of PI3K Genes

Based on the structural differences, specificities to their substrates, and differences in modes of regulation, PI3K genes are grouped into three classes (I, II, and III) (26). Class I PI3K genes control the activity of PI3K/AKT signaling and are often genetically altered in glioblastoma (29). Class II PI3K genes, while not well studied, are implicated in regulating angiogenesis and cilium function (26). Class III PI3K genes are primarily involved in the regulation of autophagy (54). In this review, we will focus on class I PI3K genes owing to their essential roles in PI3K/AKT signaling pathway and glioblastoma. The differential regulation of class I PI3K genes further divides this class into two subclasses: IA and IB. The class IA PI3K gene family consists of three highly homologous catalytic subunits *PIK3CA, PIK3CB*, and *PIK3CD* (PI3K catalytic subunit α, β, and δ) that encode p110α, p110β, and p110δ, respectively. These subunits form a complex with any of five regulatory subunits p85α, p55α (a splicing variant of p85α), p50α (a splicing variant of p85α), p85β, and p55γ, encoded by *PIK3R1, PIK3R2*, and *PIK3R3* (PI3K regulatory subunit 1, 2, and 3), respectively. Class IB PI3K is composed of one catalytic subunit p110γ encoded by *PIK3CG* (PI3K catalytic subunit γ) and two regulatory subunits: p101 encoded by *PIK3R5* (PI3K regulatory subunit 5) and p87 (also known as p84 or p87^PIKAP^) encoded by *PIK3R6* (PI3K regulatory subunit 6) (26, 55). Our recent work has revealed that class IB catalytic subunit p110γ is expressed at an undetected level in glioblastoma cells and blocking this specific subunit exhibits no cytotoxicity (56). As such, we will herein only discuss the role of class IA PI3K catalytic subunits in glioblastoma.

### PIK3CA in Glioblastoma

*PIK3CA*, frequently mutated in cancer (57), is often oncogenic (58–61); hence, much attention has been drawn on this particular PI3K subunit in many different cancers. The frequency of *PIK3CA* mutations in glioblastoma ranges from 4.3 to 26.7% due to diverse detection approaches and different sample sizes (29, 39, 40, 57, 62–65). For example, Broaderick et al. reported that 5 out of 105 glioblastoma patients harbored *PIK3CA* mutations (4.8%) (66), whereas *PIK3CA* mutations were detected in 4 samples when Sameul et al. analyzed 15 glioblastoma specimens (26.7%) (57). Genome-wide sequencing of 91 glioblastomas revealed a 6.6% mutation rate in the *PIK3CA* gene (29). However, frequencies of *PIK3CA* mutations detected by PCR amplification followed by DNA sequencing varied significantly as stated above. Based on the report from Kita et al. (62), *PIK3CA* mutations in primary (directly diagnosed as glioblastoma) or secondary (originated from low-grade gliomas) glioblastoma were 4.7% (5 out of 107) or 3.1% (1 out of 32), respectively. To date, there is no evidence showing that *PIK3CA* mutations alone are able to transform glia cells to induce the formation of glioblastoma. Additional studies investigating the role of *PIK3CA* mutants in glioblastoma are therefore needed.

Our laboratory recently analyzed the gene expression profile and clinical data from 99 recurrent glioblastomas retrieved from the TCGA database. We found that *PIK3CA* mutations had no correlation with recurrence rate. In addition, levels of PIK3CA mRNAs had no significant association with recurrence risk and recurrence-associated patient survival (56). In the same study, we knocked down PIK3CA/p110α in a panel of glioblastoma cell lines and found that loss of PIK3CA/p110α failed to both inactivate AKT and block the survival of A172, U87MG, SF295, and U251 glioblastoma cells. Our results together suggest that PIK3CA/p110α is dispensable for PI3K/AKT signaling in glioblastoma, and perhaps the progression of this deadly disease. Consistent to our results, depletion of PIK3CA using short hairpin RNAs (shRNAs) did not decrease levels of active AKT in U251 and U87MG cells and failed to inhibit the viability of U251 cells or the growth of U87MG xenograft tumors (67–69). However, inconsistent or contradictory results have been shown in some other studies. For example, Weber et al. reported that knockdown of PIK3CA/p110α blocked the survival and migration of SKMG26, D54, and primary glioblastoma cells (70). In combination with temozolomide or carmustine, small interfering RNAs (siRNAs) of PIK3CA and AKT3 substantially reduced the viability of T98G glioblastoma cells (71). Future studies should focus on clarifying the role of PIK3CA/p110α in glioblastoma using patient-derived primary glioblastoma cells in conjunction with orthotopic glioblastoma models or genetically engineered mouse glioblastoma models.

In our recent work, we tested a panel of p110α-specific inhibitors (PIK75, BYL719, MLN1117, and HS173) in glioblastoma. PIK75 and HS173 significantly inhibited the viability of glioblastoma cell lines and primary tumor cells, whereas MLN1117 and BYL719 only showed modest toxicity (56). Congruently, other studies showed that PIK75 at 100 nM blocked the growth of U87MG cells (72) or T98G cells in culture or in animals (73), whereas BYL-719 alone did not induce a remarkable growth inhibition in LN229 and U87MG cells (74). As stated earlier, p110α inhibitors often induce hyperglycemia in patients (50, 75, 76). This is consistent with our observation that p110α inhibitors are significantly toxic to astrocytes (IC50 ranging from 0.1 to 8 µM) (56). Hence, it is perhaps difficult to utilize p110α-specific inhibitors as cancer drugs due to their limited therapeutic window.

### PIK3CB in Glioblastoma

Compared to *PIK3CA*, oncogenic *PIK3CB* mutations are rare. Several studies have recently revealed that mutations at the residue D1067 (*PIK3CB*^D1067Y, D1067A, or D1067V^) display oncogenic potential, and confer resistance to erlotinib [an inhibitor of epidermal growth factor receptor (EGFR)] in non-small-cell lung cancer or decrease the sensitivity of breast cancer cells to pictilisib (also known as GDC-0941, an inhibitor of p110α/δ) (77, 78). Similarly, *PIK3CB*^A1048V^ induces tumor formation with a short latency after introduced into human embryonic kidney HA1E-M cells (79). Intriguingly, *PIK3CB*^D1067A^ and *PIK3CB*^D1067V^ have been found in two glioblastoma patients based on the TCGA genomic sequencing data of 599 glioblastoma tissues, indicative of an approximately 0.3% mutation rate. Other than oncogenic mutants described above, wild-type PIK3CB/p110β protein is able to induce tumor formation. Biochemical studies have revealed that the amino acid K342 in wild type p110β exhibits structural changes (i.e., disrupted interactions between p110β and its regulatory partner p85), which endows p110β with an unusually high transformation potential similar to the oncogenic p110α mutant p110α-N345K (80). In fact, p110β is proven to be essential for the formation of prostate and breast cancer in mouse models (81, 82) and for tumors driven by the loss of PTEN (68, 81) or GPCRs (26, 83–85).

In search of novel survival factors for glioblastoma, we recently identified 20 kinase genes important for the survival of U87MG cells (86). Through exploring the relationship of these candidates with glioblastoma recurrence using the TCGA patient data, we found that high levels of PIK3CB mRNAs correlated with high incidence and poor survival of glioblastoma recurrence (86). Compared to other class IA PI3K isoforms, PIK3CB was the only PI3K catalytic subunit that showed a strong association with recurrence rate, risk, and prognosis (56). In line with the observation that *PIK3CA* mutations and levels of PTEN exhibited no relationship with glioblastoma disease progression (56), the above results have demonstrated that PIK3CB is more important than other PI3K catalytic subunits and PTEN in the diagnosis and prognosis of glioblastoma recurrence.

Our recent work also determined the role of PI3K catalytic subunits in AKT signaling and survival of glioblastoma cells (56). First, we found that levels of p110β, but not other p110 isoforms or PTEN, strongly correlated with levels of active AKT in nine glioblastoma cell lines, eight primary glioblastoma cells, and six lines of glioblastoma stem cells. In U87MG cells that express high levels of p110β (p110β^high^), depletion of p110β inactivated AKT, whereas knockdown of other p110 proteins had no effect. In contrast, p110β knockdown failed to mitigate AKT activity in A172 cells expressing a low level of p110β (p110β^low^). These results were further substantiated by the observation that p110β-selective inhibitors are more specific in deactivating AKT than other PI3K isoform-selective inhibitors. These results demonstrate that p110β dictates AKT activation and promotes cell survival in p110β^high^ glioblastoma cells. This conclusion is further supported by the results from other studies. Zhao et al. showed that the p110β-specific inhibitor TGX-221 significantly decreased the levels of phosphorylated AKT at both S473 and T308 (pAKTS473 and pAKTT308) in U87MG cells, whereas the p110α inhibitor PIK75 and p110δ inhibitor CAL101 only induced a reduction of pAKTT308, but not pAKTS473 (72). This difference may explain why SP600125 (an inhibitor of c-Jun N-terminal kinases) only synergizes with TGX-221, but not PIK75 or CAL101, to inhibit cell viability and tumor growth (72). Chen et al. (67) and Pu et al. (87) also reported that shRNAs of PIK3CB, but not PIK3CA, inactivated AKT and decreased cell viability of U251 cells. More importantly, depletion of PIK3CB substantially blocked the growth of U251 xenografts in mice. Similarly, Wee et al. (68) and Millan-Ucles et al. (88) showed that siRNAs or shRNAs of PIK3CB, but not shRNAs of PIK3CA, inhibited the growth of U87MG cells.

We exploited a panel of PI3K isoform-selective inhibitors and tested their activities on glioblastoma cell viability (56). The p110β inhibitor TGX-221 and GSK2636771 mitigated the proliferation of p110β^high^ U87MG and SF295 cells while having no effect on p110β^low^ A172 and LN229 cells. The p110α inhibitor PIK75 showed a strong cytotoxicity to all glioblastoma cell lines tested, whereas another p110α inhibitor BYL719 failed to display selective growth inhibition in p110α^high^ cells. To provide a possible explanation for the unusually high toxicity of PIK-75, we monitored activity of AKT and extracellular signal-regulated kinase (ERK) and found that PIK-75 inhibited both AKT and ERK, suggesting that this particular p110α inhibitor is not a selective inhibitor of PI3K/AKT. By contrast, the p110β inhibitor TGX-221 only attenuated the activity of AKT, but not ERK. Hence, p110β inhibitors are more selective in affecting PI3K activities in glioblastoma. Relevant to clinical applications, IC50s of p110β inhibitors in human astrocytes are relatively high (100.4 and 291 µM, respectively). In stark contrast, other PI3K inhibitors robustly block astrocyte growth with IC50s ranging from 0.1 to 21.2 µM. Given this low cytotoxicity to normal astrocytes, selectively targeting PIK3CB/p110β is expected to have minimal side effects, thereby representing an appealing approach in treating glioblastoma.

### PIK3CD in Glioblastoma

A PIK3CD/p110δ inhibitor idelalisib had achieved a plausible clinical outcome in treating chronic lymphocytic leukemia (CLL) (89). While PIK3CD/p110δ has been well studied in blood cancers, little is known about the role of this subunit in glioblastoma. We have probed different glioblastoma cells with a PIK3CD/p110δ antibody (56). We found that this subunit was differentially expressed in glioblastoma cells and its expression levels did not correlate with AKT activation. Moreover, knockdown or inhibitors of PIK3CD/p110δ failed to inhibit AKT and cell viability. Our results suggest that PIK3CD/p110δ plays a dispensable role in the disease progression of glioblastoma. This conclusion is also supported by studies from Jones et al. (90), Holland et al. (73), and Zhao et al. (72). In these studies, treatment of U87MG, T98G, or LN18 cells with PIK3CD/p110δ inhibitors CAL101 or IC87114 displayed no or negligible cytotoxicity.

However, Schulte et al. have shown that expression of PIK3CD/p110δ, but not other PI3K catalytic subunits, is significantly enhanced in BS153 glioblastoma cells with acquired resistance to the EGFR inhibitor erlotinib (91). siRNA of p110δ restores erlotinib sensitivity in BS153 cells. In another study, siRNA-mediated knockdown of p110δ mitigates the migration of U87MG cells, whereas siRNAs of p110α and p110β exhibit no activity (92). Together, these results suggest that PIK3CD/p110δ regulates the migration, but not the survival, of glioblastoma cells. Nonetheless, the role of this subunit in glioblastoma and whether targeting PIK3CD/p110δ is an effective treatment for glioblastoma require further research.

### Divergent Roles of Class I PI3K Genes in Normal and Malignant Tissues

A growing body of information strongly suggests that the four PI3K catalytic subunits are not functionally redundant in normal and malignant tissues. For example, insulin signaling often requires p110α, but not p110β, suggesting that blockade of p110α may be more harmful to normal tissue than inhibiting p110β (81, 93–96). Indeed, p110α inhibitors often induce hyperglycemia in patients (50, 75, 76). Two other PI3K catalytic subunits, p110γ and p110δ, are highly expressed in immune cells and have been shown to be more involved in maintaining homeostasis of the immune system and in the development of blood malignancies (97–100). The p110δ inhibitor, idelalisib (CAL-101), has been recently approved by the Food and Drug Administration to treat relapsed CLL, and this drug has achieved an overall response in more than 70% of patients in a phase I clinical trial (89). Furthermore, a combination of idelalisib and rituximab (cytotoxic anti-CD20 antibody) resulted in a 95% overall response, including a 19% complete response of relapsed CLL patients (101).

**Figure 1:**
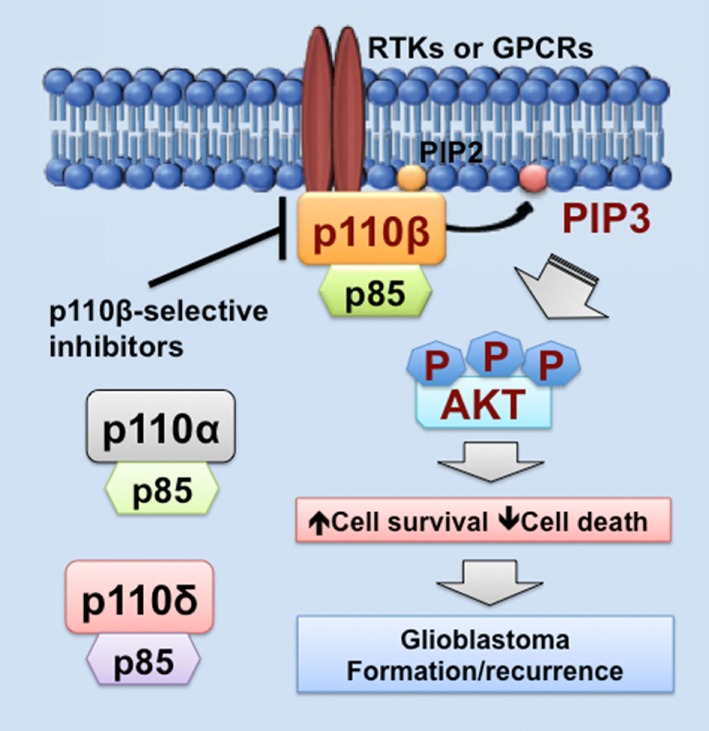
PIK3CB/p110β in glioblastoma. This figure illustrates a PIK3CB/p110β-dictated survival pathway in glioblastoma. Receptor tyrosine kinases (RTKs) or G protein-coupled receptors (GPCRs) selectively activates PIK3CB/p110β (but not PIK3CA/p110α or PIK3CD/p110δ), leading to production of phosphatidylinositol-3,4,5-triphosphate (PIP3) and subsequent phosphorylation of AKT. The activation of this signaling pathway promotes cancer cell survival while inhibiting cell death, the consequences of which are drug resistance and tumor recurrence. Hence, PIK3CB/p110β promotes glioblastoma disease progression and targeting PIK3CB/p110β using selective inhibitors represents a novel and effective approach to treating glioblastoma.

As we discussed above, PIK3CB/p110β is more important in cell survival than other PI3K catalytic subunits in glioblastoma. Similar results have also been shown in colorectal and breast cancer (68, 81, 100). One possible explanation is that PI3K catalytic subunits are differentially expressed in these cancers. Another possible reason is that upstream activators of PI3K catalytic subunits differ in these cancers. In fact, RTKs often signal through p110α, p110β, or p110γ, whereas GPCRs selectively activate p110β (26, 83, 102, 103). Herein, we speculate that GPCRs preferentially activate PIK3CB/p110β, but not other PI3K catalytic subunits, in glioblastoma expressing high levels of PIK3CB/p110β. This preferential activation of PIK3CB/p110β results in sustained cell survival, which promotes glioblastoma progression by inducing drug resistance and leading to tumor recurrence (Figure 1). Hence, PI3K catalytic subunits have different assignments in normal and malignant tissues, highlighting the importance of selectively targeting one PI3K catalytic subunit to treat cancer.

## Concluding Remarks

Research described above has demonstrated that PI3K catalytic subunits are not functionally redundant in glioblastoma. In particular, research from our group strongly suggests that PIK3CB/p110β is a dominant PI3K catalytic subunit that dictates PI3K/AKT signaling, thereby becoming a selective survival factor for glioblastoma. Future research will focus on elucidating the molecular mechanisms underlying the selective activation of p110β/AKT signaling in p110β^high^ glioblastoma. These details at the molecular level will shed light on the design and production of PIK3CB/p110β-selective inhibitors with stronger inhibition of glioblastoma and less toxicity to normal brain tissues. Taken together, PI3K catalytic subunits play divergent roles in glioblastoma, underscoring the importance and necessity of selectively targeting one PI3K subunit to treat glioblastoma.

## Author Contributions

ZS: conception of this review article, preparation of figures, and manuscript writing. KP: preparation of chapter I and manuscript editing. RV: preparation of chapter I and Table 1 and manuscript editing.

## Conflict of Interest Statement

The authors declare that the research was conducted in the absence of any commercial or financial relationships that could be construed as a potential conflict of interest.
